# Xingnao Kaiqiao Acupuncture Method Combined with Temporal Three-Needle in the Treatment of Acute Ischemic Stroke: A Randomized Controlled Trial

**DOI:** 10.1155/2022/8145374

**Published:** 2022-06-29

**Authors:** Zhongtao Song, Qingyuan Huang, Yuxia Guo, Xiaodong Song, Xuan Zhang, Huajun Xiao

**Affiliations:** Meizhou People's Hospital, Meizhou 514031, Guangdong, China

## Abstract

**Objective:**

The objective of this study was to investigate the difference between the clinical effectiveness of two acupuncture methods in the treatment of acute ischemic stroke (AIS) and provide more evidence-based medical evidence of acupuncture's effectiveness in stroke rehabilitation.

**Methods:**

We conducted an outcome assessor-and data analyst-blinded, randomized, and controlled trial. Seventy-two participants were randomly allocated to the observation group and control group with a 1 : 1 allocation ratio by the generating of a random number table.The observation group received the “Xingnao kaiqiao” acupuncture method combined with “Temporal three needles,” and the control group received conventional acupuncture “Scalp acupuncture” combined with the traditional “body acupuncture” method. The acupuncture treatment was performed once per day for one week by trained acupuncturists. Both groups underwent secondary prevention of cerebral infarction and received a 3-months' followup. After a 1-week acupuncture intervention, the changes of NIHSS (National Institutes of Health Stroke Scale) scores, Percent Change and Absolute Change of NIHSS scores, MBI (Modified Barthel Index), and the rate of MBI ≥ 80 in two groups were observed. After 3 months' followup, the mRS (Modified Ranking Scale) and the clinical efficacy of the two groups were compared.

**Results:**

The apparent efficiency rate of the observation group was 63.9%, higher than 19.4% of the control group, and the difference was significant (*P* < 0.05). After treatment, NIHSS scores, Percent Change, and Absolute Change of NIHSS scores in the observation group had a significant reduction than the control group (all *P* < 0.05). MBI in the observation group increased significantly more than in the control group (*P* < 0.05), but the rate of MBI ≥ 80 in the two groups was not significantly different (*P* > 0.05). After 3 months' of followup, the mRS score frequencies of the observation group were not statistically different from the control group (*P* > 0.05). The rate of mRS scores of 0–1 in the observation and control group were 55.6% and 38.9%, and there was no significant difference either (*P* > 0.05).

**Conclusion:**

Compared with “Scalp acupuncture” combined with “body acupuncture,” “Xingnao kaiqiao” acupuncture method combined with “Temporal three-needle” had superiority in the improvement of neurological deficit, potential functional disability, and score of basic activities of daily living. As to the independent rate to basic activities of daily living and good prognosis of 3 months, there were no statistical differences.

## 1. Introduction

Ischemic stroke, with the common impairments that include motor and sensory loss or alteration [1], seriously affects the quality of life of patients and causes a tremendous burden to the whole society. Acute ischemic stroke is the most important stage to develop effective early interventions to improve the prognosis for patients. Currently, administration of recombinant tissue plasminogen activator (rtPA) within 3 h of stroke onset improves clinical outcomes, large vessel occlusions that fail to open with intravenous (IV) rtPA alone are candidates for endovascular revascularization [2]. Endovascular therapy coupled with mechanical thrombectomy is the preferred choice to improve the reperfusion rate [3]. However, both therapies are severely restricted by the treatment time window [4] and the related risk of bleeding concerns patients and their families greatly.

Acupuncture, widely used for thousands of years in China [5], play an important role in the rehabilitation treatment for the patients with ischemic stroke. Up to now, there are two main representative methods involving scalp acupuncture combined with traditional body acupuncture and “Xingnao kaiqiao” (restoring consciousness and inducing resuscitation) acupuncture technique [6]. Intending to investigate the difference between the effectiveness of two acupuncture methods in the treatment of acute ischemic stroke, we conduct an outcome assessor-and data analyst-blinded, randomized, and controlled trial.

## 2. Clinical Data

### 2.1. General Data

All participants were collected from inpatients from the Department of Chinese Medicine and Neurology of Meizhou People's Hospital, from May 1, 2019 to January 1, 2021. A total of 72 patients were included and they were randomly allocated to the observation group and control group with a 1 : 1 allocation ratio by the generating of a random number table. There were 36 cases in the observation group, aged from 33 to 87 years old, averagely (67.28 ± 13.34) years old, with the time since stroke onset 0.25 to 14 days, the Median (*P*_25_, *P*_75_) of which is 2(1–3.75) days. In the control group, 36 cases aged from 45 to 87, averagely (66.17 ± 10.69) years old, with the time since stroke onset 0.25 to 14 days, the Median (*P*_25_, *P*_75_) of which is 2(1–4) days. Regarding the age and disease duration of the subjects, there was no significant difference between groups, which made them comparable. The protocol was approved by the ethics committee of Meizhou People's Hospital on April 9, 2019.  Table 1 shows the comparison of general data between the two groups.

### 2.2. Diagnosis Criteria

The diagnosis criteria of traditional Chinese medicine were in reference with the standard for diagnosis and efficacy evaluation of stroke (Trial); the diagnosis criteria of western medicine were in reference with the diagnostic criteria of acute ischemic stroke according to the Chinese guidelines for diagnosis and treatment of acute ischemic stroke 2018 [1].

### 2.3. Inclusion Criteria

Patients are eligible for study inclusion if they: (1) were aged 18–80 years; (2) met the diagnostic criteria of acute ischemic stroke; (3) the onset was less than 2 weeks and the vital signs were stable; (4) there were obvious physical activity, language dysfunction and other symptoms; (5) Glasgow coma scale (GCS) > 8; (6) First onset or previous stroke without neurological deficit; (7) were willing to accept and complete the course of treatment; (8) patients or family members informed consent to accept the clinical trial.

### 2.4. Exclusion Criteria

Patients will be excluded if they have any one of the following: (1) the patient was diagnosed with hemorrhagic stroke; (2) the patient had taken thrombectomy or intravenous thrombolysis; (3) the patient had serious cardiovascular, liver, kidney, or hematopoietic system disease; (4) the patient had an infection of the respiratory system, urinary system, digestive system, or other parts of the body; (5) the patient was a pregnant or lactating woman; (6) the patient had psychotic, dementia, or others problems that unable to cooperate with treatment; (7) the patient had neurological deficit or motor dysfunction caused by past or other reasons.

### 2.5. Withdrawal or Dropout Criteria

Participants will be terminated to continue this trial according to the following criteria:(1) the participants who have a deteriorated condition or have had a serious adverse event; (2) the participants who suffer from certain complications or specific physiological changes during the study may not be appropriate to continue; (3) the participants who fail to complete the course of treatment.

## 3. Treatment Methods

All patients were given second prevention of ischemic stroke in accordance with the Chinese guidelines for the diagnosis and treatment of acute ischemic stroke, to monitor and control the high blood pressure, high cholesterol, sugar metabolic disorder, and risk factors such as diabetes.

### 3.1. Observation Group

Received “Xingnao kaiqiao” acupuncture method combined with “Temporal three-needle,” the parameters of which were set as follows:

1. Acupoint: Neiguan (PC6,bilateral), Renzhong (DU26, unilateral), Sanyinjiaog (SP6, unilateral), Temporalthree-needle (unilateral, the affected side).
2. The point location has referred the nomenclature and location of acupuncture points (GB/T12346-2006) [7], temporal three-needle is one of Jin's three-needle therapy systems created by Professor Jinrui, the temporal I needle is located on the hairline 2 cun above the apex of the ear; the temporal II needle and the temporal III needle are located 1 cun from the temporal I needle on either side in the horizontal direction [8].
3. Acupuncture manipulation: participants were asked to adopt a supine position, then Neiguan (PC6) was punctured bilaterally to a depth of 0.5–1.0 cun and stimulated with the reducing method by lifting and thrusting with simultaneous twirling manipulation for 1 min. After this, Renzhong (DU26) was punctured obliquely toward the nasal septum to a depth of 0.3–0.5 cun with bird-pecking needling until the eyes became wet or developed tears. Subsequently, Sanyinjiaog (SP6) was punctured on the affected side obliquely along with the medial border of the tibia and at a 45° angle to the skin to a depth of 0.5–1.0 cun, with lifting and thrusting reinforcing manipulation, thrusts with heavy strength and lifting with gentle strength until calf twitch for 3 times. At last, the temporal three-needle was punctured in sequence at an approximately 15-degree angle to a depth of 0.8–1.2 cun, with the needle rotated for at least 200 revolutions per minute for 1 minute.

### 3.2. Control Group

“Scalp acupuncture” combined with “body acupuncture” method was received once per day for 1 week. The parameters for “scalp acupuncture” combined with the “body acupuncture” acupuncture method are set as follows:

1. Acupoints: the motor area of Jiao's scalp acupuncture (unilateral, the affected side), Jianyu (LI15, unilateral), Quchi (LI11, unilateral), Waiguan (SJ5, unilateral), Hegu (LI4, unilateral), Yanglingquan (GB34, unilateral), Zusanli (ST36, unilateral), Fenglong(ST40, unilateral), Kunlun (BL60, unilateral). Acupoint location has referred the Nomenclature and Location of acupuncture Points (GB/T12346-2006) [7] The motor area of Jiao's scalp acupuncture is a line starting from a point (known as the upper point of the motor area) 0.5 cm posterior to the midpoint of the anterior-posterior midline of the head and stretching diagonally to the juncture between the eyebrow-occipital line and the anterior border of the corner of the temporal hairline, which is indistinct. Draw a vertical line upwards from the middle point of the zygomatic arch to the eyebrow-occipital line; the intersection of the two lines is the projection of the motor area [9]. The motor area of the cerebral infarction lesion's side is selected as the site for acupuncture treatment, and the paralytic side is selected for the other acupoints.
2. Acupuncture manipulation: Participants were asked to adopt the supine position, then needles were manually inserted at an approximately 15-degree angle to a depth of 0.8–1.0 cun respectively along the line of the motor area on the scalp. The needles were rotated for at least 200 revolutions per minute for 1 minute. For other acupoints, needles were punctured to a depth of 0.8–1.0 cun, then the lifting, thrusting, and twirling manipulation was performed until the sensation of Deqi. All the needles were retained for 30 minutes.

For acupuncture therapy mentioned in both groups, disposable stainless steel needles (size 0.30 mm × 40 mm, Tony brand, manufactured by Suzhou Medical Appliance) were used after routinely disinfecting the local skin of acupoints with 75% alcohol cotton swabs. The acupuncture treatment was performed once per day for one week by a trained acupuncturist with more than 3 years of clinical experience.

After the 1-week treatment observation, all patients will start an additional 3-month followup period. Because of the specificity of stroke patients' recovery, patients from both groups would need to attend rehabilitation treatment during the followup period.

## 4. Therapeutic Effect Observation

### 4.1. Observation Indicators

#### 4.1.1. NIHSS (National Institutes of Health Stroke Scale)

The National Institutes of Health Stroke Scale, a 15-item scale, allows consistent reporting of neurological deficits in acute-stroke studies, which contains quantified basic neurological examination. The NIHSS provides an ordinal, nonlinear measure of acute stroke-related impairments by assigning numerical values to various aspects of neurological function [10]. NIHSS scores range from 0 to 42, with higher scores indicating a more severe neurological deficit. The NIHSS has a high reliability to observe after only a few hours of training, is easy and quick to assess and is a valid measure of stroke severity. In this study, the NIHSS was assessed at admission, after the interventional period (at 1 week). Percent Change NIHSS was defined as [admission NIHSS score-1-week NIHSS score] × 100/admission NIHSS score]. Absolute Change NIHSS was defined as [admission NIHSS score-1-week NIHSS score].

#### 4.1.2. Modified Ranking Scale (mRS)

The ranking scale was a scale introduced by Dr. John Rankin in 1957 and modified to its current form by Charles Warlow in the 1980s, which has grown in popularity for almost all acute stroke trials [11, 12]. The mRS is now the most commonly used functional measure in stroke trials and has been the primary or coprimary outcome in most recent large-scale stroke trials [10]^.^ The mRS has many potential strengths, and it is well accepted by both patients and assessors, with a nonstandardized interview taking around 5 minutes to complete. There are seven potential scores on the mRS (0–6), describing a full range of stroke outcomes from perfect health without symptoms to death. In this trial, the mRS was assessed when the followup period ended (at 3 months). An mRS score of 0–1 is considered as a good recovery.

#### 4.1.3. Modified Barthel Index (MBI)

The Barthel Index is a scale that measures ten basic aspects of daily life activities related to self-care and mobility [13]. For the Chinese MBI version, the ten items are continence of bowels and bladder, feeding, dressing, entering and leaving a toilet, grooming, bathing, moving from a wheelchair to a bed and returning to a wheelchair, walking on a level surface for 45 m, and ascending and descending stairs. Each item (activity) can be divided into five levels; each level represents a different degree of independence, the lowest level being 1 and the highest being, and the higher the level, the greater the independence. The total possible score is 100. Subjects with an independent living ability score 80 or more; lower scores represent assisted or totally dependent living [14]. The MBI was assessed at admission, and after the interventional period (at 1 week).

### 4.2. Effect Criteria

The therapeutic effect evaluation standard was set as follows:

  Basically cured:NIHSS score reduction ≥90%, and mRS = 0
  Markedly effective: NIHSS score reduction ≥46%, but<90%, and mRS = 1,2,3
  Effective: NIHSS score reduction ≥18%, but <46%
  Invalid: NIHSS score reduction <18%
  The Sum of basically cured and markedly effective cases were calculated for apparent efficiency rate.

### 4.3. Statistical Methods

SPSS 22.0 software (SPSS Inc., Chicago, IL, USA) was employed for all statistical analyses. Normal distribution quantitative data were expressed as $\bar{x}\pm s$ and analyzed by independent *t*-test or paired *t*-test, non-normal distribution quantitative data were expressed as *M* (*P*_25_, *P*_75_) and analyzed by Mann–Whitney test. The enumeration data were expressed as counts (percentages). The rank-sum test was adopted to analyze categorical data, and *P* < 0.05 indicated that the difference was statistically significant.

## 5. Results

### 5.1. Comparison of the Clinical Effect

The apparent efficiency rate of the observation group was 63.9%, higher than19.4% of the control group, and the difference was significant (*P* > 0.05). The overall curative effect of the observation group was better than that of the control group (*P* > 0.05), as shown in Table 2.

### 5.2. Comparison of NIHSS Score (before and after Treatment) and Percent Change NIHSS

Before treatment, there was no statistical significance in NIHSS scores between the two groups (*P* > 0.05). After treatment, NIHSS scores in both groups were reduced than before (both *P* < 0.05), and the reduction in the observation group was more obvious compared with the control group. There was a statistical significance difference between the two groups (*P* < 0.05). In a comparison of Percent Change and Absolute Change of NIHSS in the observation and control group, a significant difference was observed (*P* < 0.05) as shown in Tables 3 and 4.

### 5.3. Comparison of MBI (before and after Treatment) and the Percent of MBI ≥ 80 after Treatment

Before treatment, there was no statistical significance in MBI between the two groups (*P* > 0.05), After treatment, MBI in both groups were increased more than before, but the improvement in the observation group was more obvious and there was statistical significance between groups (*P* < 0.05). Although the percent of MBI ≥ 80 in the observation group was higher than that in the control group, but the difference was not statistically significant (*P* > 0.05) as shown in Table 5.

### 5.4. Comparison of mRS Score Frequencies after 3-Month Followup

After 3-month followup, mRS score frequencies of the observation group were not statistically different from the control group (*P* > 0.05). The rate of mRS score of 0–1 in the observation and control groups were 55.6% and 38.9%, and there was no significant difference (*P* > 0.05), although the proportion in the observation group had possible higher trend than that in the control group as shown in Table 6.

## 6. Discussion

The NIHSS within 1 week satisfies the requirements for a surrogate end point and may be used as a primary outcome measure in trials of acute treatment for ischemic stroke [15]. It measures neurological deficit rather than the functional outcome. The early NIHSS is a very useful primary outcome measure in clinical trials testing the effect of new therapeutic interventions for ischemic stroke. The 7-day relative neurological improvement on NIHSS can predict 90-day functional outcomes after therapeutic interventions better than the 24-hour relative neurological improvement [16]. Percent Change NIHSS and Absolute Change NIHSS have powerful abilities to predict the functional outcome of the patients with stroke, and Percent Change NIHSS has the better predictive ability [17]. In this study, the NIHSS score, Percent Change NIHSS, and Absolute Change NIHSS of the observation group improved more obviously than the control group, which marked a good outcome. Barthel Index is the most commonly used functional measure in stroke-rehabilitation settings and the second most commonly used functional outcome measure across stroke trials. Most Chinese researchers usually use a slightly modified edition of the BI to adapt to China's condition without changing the validity of this tool and the accuracy of responses. It is usually believed that subjects with MBI > 80 are generally independent. As Results showed previosuly, after a 1-week acupuncture intervention independent (to basic activities of daily living) rate of participants in the observation group was not significantly higher than the control group. Although the original Rankin Scale was not originally intended as an assessment for clinical trials, a slightly modified version of Rankin's eponymous scale was used as an end point in the first multicenter stroke trial (the UK TIA study) [18]. Recent studies have quantified the responsiveness of the mRS and proven excellent construct and convergent validity of the scale. Now, the mRS has grown to be the most prevalent functional outcome measure in contemporary stroke trials and has been used in several landmark studies [19, 20]. As the results shown, the observation group failed to make a better recovery (3 months after acupuncture intervention) than the control group.

There are many acupuncture techniques in the treatment of ischemic stroke. Scalp acupuncture has shown a remarkable treatment efficacy on motor dysfunction in patients with stroke in China, especially in the motor area of Jiao's scalp acupuncture, which is the most widely used treatment [21]. Many studies have shown that scalp acupuncture has a remarkable treatment efficacy on motor dysfunction in stroke patients in China. In clinical practice, scalp acupuncture often combines with traditional body acupuncture. The traditional body acupuncture treatment of stroke patients mostly selects acupoints on Yang meridians, which has the theoretical basis “To treat paralysis, take “Yangming Meridian alone” recorded in *Huangdi Neijing (Inner Canon of the Yellow Emperor)*. The “Xingnao kaiqiao” acupuncture technique was developed by Xue-min Shi in 1972 for the treatment of stroke [22], especially ischemic stroke, based on traditional Chinese medicine theory, modern medicine theory, and clinical treatment experience over 30 years and made a great contribution [23]. It is a special acupuncture therapy system, that contained its own theory about the cause and pathogenesis of stroke, the principle of treatment, the formula of acupoint selection, and the quantitative standard of acupuncture manipulation. It was totally different from the traditional acupuncture therapy. According to the “Xingnao kaiqiao” acupuncture theory, the most important pathogenesis of stroke is the hidden of mind induced by the occlusion of cerebral orifices. The principle of acupuncture treatment is based on “restoring consciousness and inducing resuscitation, nourishing the liver and kidney,” and “unblocking the meridians” as the supplement. Acupoints selected were mainly on Yin Meridians, and corresponding manipulations were quantitatively prescribed for the optimal stimulus amount and the objective response of the patients. Evidence from previous studies [24–26] has shown that the Xingnao Kaiqiao acupuncture achieved satisfactory treatment effects on stroke. But the contrast study of the curative effect between the two major acupuncture methods is rare. In order to compare the advantages of the two acupuncture methods in neurological deficit, quality of life, and good prognosis, we conducted this study.

Results of this study confirmed that the “Xingnao kaiqiao” acupuncture method combined with the “Temporal three-needle” had a better clinical efficacy on AIS than the conventional acupuncture method, as previous studies [27, 28] had shown. And, according to the results, the NIHSS score of the observation group significantly improved than the control group after a 1-week acupuncture intervention. Both Absolute Change and Percent Change of NIHSS in observation group were better than that in the control group which indicated the potential functional disability of participants got improved more effectively in the observation group. The “Xingnao kaiqiao” acupuncture method combined with the “Temporal three-needle” had advantages in improving neurological deficits and potential functional disability of patients with AIS. The MBI score of the observation group was significantly higher than the control group after a 1-week acupuncture intervention, which indicated the basic ability of daily living of patients in the observation group got improved better than the control group, but the ratio of MBI ≥ 80 in the observation group was not significantly higher than that in the control group (*P* > 0.05). The independent (to basic activities of daily living) rate of patients in the two groups was not significantly different. The “Xingnao kaiqiao” acupuncture method combined with the “Temporal three-needle” had advantages in improving activities of daily living of patients with AIS, but failed to increase their independent rate of them. Based on the results mentioned above, mRS score frequencies of the observation group were not statistically different from the control group, so as the proportion of mRS score of 0–1. In other words, there was no significant difference in the prognosis between the two groups when the 3-month followup ended.

Modern research studies have shown that the “Xingnao Kaiqiao” acupuncture method has an obvious brain protective effect, of which mechanism may be interfering inflammatory response in the brain after cerebral ischemia reperfusion [29], regulating the opening of brain KATP channel, reducing neuronal apoptosis [30], and starting the endogenous protection mechanisms from the overall level. Furthermore, the “Xingnao Kaiqiao” acupuncture improved the cognitive function of patients, and its mechanism may be related to down regulating the levels of serum MMP-2 and MMP-9 and increasing the level of IGF-1 [31]. Based on the above advantages, the “Xingnao Kaiqiao” acupuncture method has possibility to achieve a better curative effect. This study verified the therapeutic effect of the “Xingnao Kaiqiao” acupuncture method on patients with AIS, such as the improvement of neurological impairment, potential functional disability, and the basic ability of daily living but not including good prognosis after 3 months of followup. The clinical efficiency of the conventional acupuncture method is mainly reflected in the long term prognosis, the possible reason of which estimated by us was that the method was summarised and refined based on evidence of long term clinical practice of so many acupuncturists. The mechanism of acupuncture in the treatment of ischemic stroke is not clear yet, more in-depth research is needed.

## 7. Conclusion

Compared with “Scalp acupuncture” combined with “body acupuncture,” the “Xingnao kaiqiao” acupuncture method combined with “Temporal three-needle” had superiority in the improvement of neurological deficit, potential functional disability, and score of basic activities of daily living. As to the independent rate to basic activities of daily living and good prognosis of 3 months, there were no statistical differences.

## Figures and Tables

**Table 1:** Comparison of general data between two groups.

Groups	*n*	Gender		Age (years)			Time since stroke onset (days)		
		Male	Female	Youngest	Eldest	Average ($\bar{x}\pm s$)	Shortest	Longest	*M* (*P*_25_, *P*_75_)

Observation	36	19	17	33	87	67.28 ± 13.34	0.25	14	2(1–3.75)
Control	36	21	15	45	87	66.17 ± 10.69	0.25	14	2(1–4)
Statistical value		0.225^a^				0.390^b^			0.034^c^
*P*-value		0.635				0.698			0.973

a. *χ*^2^-value; b. *t*-value; c. *Z*-value.

**Table 2:** Comparison of the clinical effect.

Groups	*n*	Basically cured	Markedly effective	Effective	Invalid	Apparent efficiency rate

Observation	36	4(11.1%)	19(52.8%)	3(8.3%)	10(27.8%)	63.9%
Control	36	4(11.1%)	3(8.3%)	11(30.6%)	18(58.0%)	19.4%
Statistical value		2.701^a^				14.629^b^
*P*- value		0.007				0.000

a.*Z-*value; b.*χ*^2^-value.

**Table 3:** Comparison of NIHSS score (before and after treatment).

Groups	*n*	NIHSS(before treatment)		NIHSS(after treatment)	
		*M* (*P*_25_, *P*_75_)	Range	*M* (*P*_25_, *P*_75_)	Range

Observation	36	4(3–7)	1–15	2(1–3.75)^b^	0–13
Control	36	5(3–9)	1–16	4(2–7)^a^	0–16
*Z*- value		0.958		2.928	
*P*- value		0.338		0.003	

**Table 4:** Comparison of percent change and absolute change of NIHSS.

Groups	*n*	Percent change NIHSS				Absolute change NIHSS
		≤25%	>25%, and ≤50%	>50%, and ≤75%	>75%	*M*(min, max)

Observation	36	4(11.1%)	19(52.8%)	3(8.3%)	10(27.8%)	2(−4,11)
Control	36	4(11.1%)	3(8.3%)	11(30.6%)	18(58.0%)	1(−3,6)
*Z*- value		−3.312				−2.780
*P*- Value		0.001				0.005

**Table 5:** Comparison of MBI (before and after treatment) and the percent of MBI ≥ 80 after treatment.

Groups	*n*	MBI (before treatment)		MBI (after treatment)		Percent of MBI ≥ 80
		*M* (*P*_25_, *P*_75_)	Range	*M* (*P*_25_, *P*_75_)	Range	

Observation	36	60(35–75)	20–100	85(65–100)	20–100	58.3%
Control	36	60(36.25–80)	20–100	65(40–85)	15–100	38.9%
Statistical value		0.384^a^		−2.273^a^		2.724^b^
*P*- value		0.701		0.023		0.099

a.Z-value; b. *χ*^2^-value.

**Table 6:** mRS score frequencies after 3-month followup.

Groups	*n*	0	1	2	3	4	5	6	The rate of 0–1

Observation	36	8 (22.2%)	12 (33.3%)	7 (19.4%)	4 (11.1%)	5 (13.9%)	0 (0%)	0 (0%)	55.6%
Control	36	7 (19.4%)	7 (19.4%)	7 (19.4%)	10 (27.8%)	4 (11.1%)	1 (2.8%)	0 (0%)	38.9%
Statistical value		1.181^a^							2.006^b^
*P*- value		0.238							0.157

a.*Z*-value; b.*χ*^2^-value.

## Data Availability

The data used to support the findings of this study are available from the corresponding author upon request.
